# New individualized aflibercept treatment protocol for branch retinal vein occlusion with macular edema

**DOI:** 10.1038/s41598-023-28533-z

**Published:** 2023-01-27

**Authors:** Hidetaka Noma, Kanako Yasuda, Akitomo Narimatsu, Masaki Asakage, Masahiko Shimura

**Affiliations:** Department of Ophthalmology, Hachioji Medical Center, Tokyo Medical University, 1163, Tatemachi, Hachioji, Tokyo 193-0998 Japan

**Keywords:** Medical research, Molecular medicine

## Abstract

We evaluated the long-term (24-month) efficacy of a novel individualized treatment protocol with 2 mg aflibercept for treatment-naive BRVO with macular edema. Each patient received an initial aflibercept injection and was then examined every 2 weeks until recurrence of edema. At recurrence, each patient received a second injection of aflibercept. The period of efficacy was defined as the time between the first and second injections. Subsequently, each patient was examined and re-injected with aflibercept at their personalized treatment interval, which was defined as 1 week shorter than the period of efficacy. Thirty-seven eyes of 48 patients showed recurrence after the initial injection. The mean period of efficacy was 92.5 ± 40.8 days, and the mean number of visits before recurrence, 7.6 ± 2.9. The mean 24-month best corrected visual acuity (BCVA) was significantly better than the mean baseline BCVA but significantly worse than the best BCVA during the period of efficacy. The mean gain of BCVA at 24 months was 0.07 ± 0.18 logMAR. The mean 24-month central macular thickness (CMT) was significantly lower than the mean baseline CMT but showed no difference from the mean best CMT (*p* = 0.060). The mean total number of visits during the 24 months was 15.8 ± 3.4. We conclude that the individualized treatment protocol that was based on the period of efficacy in treatment-naïve BRVO eyes with macular edema achieved satisfactory long-term visual outcome.

## Introduction

Branch retinal vein occlusion (BRVO) occurs mainly in older adults. According to a pooled analysis of data from international studies in adults aged 30 years and above, the age- and sex-standardized prevalence of BRVO is 4.42 cases per 1000 people; furthermore, prevalence varies between races and ethnicities but is similar in men and women^1^.

One of the main complications of BRVO is macular edema, which can result in blindness. Treatments for BRVO-related macular edema include intravitreal injections of an anti-vascular endothelial growth factor (VEGF) agent. One study that compared the anti-VEGF drug ranibizumab in monotherapy, laser in monotherapy, and a combination of the two treatments found an improvement in best-corrected visual acuity (BCVA) of 17.3 letters in the combined treatment group, 15.5 in the ranibizumab only group, and 11.6 in the laser only group^2^. Real-world data showed a visual prognosis of a 13.7-letter gain after anti-VEGF treatment with ranibizumab, with 5.8 injections being required per 2-year period^3^. Another study also showed improvements in BCVA with ranibizumab but found that patients may need as many as 10 to 11 injections over a 2-year follow-up^4^. Repeated injection of intravitreal anti-VEGF drugs sometimes causes adverse effects, including ocular pain, ischemic retinopathy, and endophthalmitis^5^, and is also expensive. In addition, some patients respond well to treatment whereas others show poor or no treatment response^6^. For this reason, authors have suggested that treatment protocols should be individualized^4^. When considering individualized protocols, response to anti-VEGF drugs is the most important factor and should be clarified in each eye with BRVO and macular edema. Therefore, this 24-month study aimed to investigate an individualized anti-VEGF treatment regimen with intravitreal injections of aflibercept in untreated patients with macular edema due to BRVO.

## Results

### Demographics of patients

The study included a total of 48 eligible eyes with BRVO and macular edema in 48 patients. The mean length of time from the first symptoms to the first ophthalmological examination was 41.9 ± 27.4 days (range, 7–90 days). In the 24-week study period, macular edema recurred in 37 eyes of 37 patients (77.1%; 12 men, 25 women). In the 37 patients with recurrence, none of whom dropped out of the study, the mean age was 68.3 ± 9.5 years; mean baseline BCVA, 0.37 ± 0.24 logMAR; and mean CMT, 656 ± 185 mm. In contrast, in the 11 patients (22.9%; 7 male, 4 female) without recurrence the mean age was 64.5 ± 8.5 years; mean baseline BCVA, 0.35 ± 0.31 logMAR; and mean CMT, 520 ± 176 mm. The initial parameters showed no significant difference between the 2 groups (age,* p* = 0.228; BCVA;* p* = 0.743; duration of preclinical period, *p* = 0.121; Table 1). CMT showed significant difference between the 2 groups (*p* = 0.036; Table 1).

**Table 1:** Characteristics of patients with untreated macular oedema due to branch retinal vein occlusion before starting treatment with intravitreal injections of the anti-vascular endothelial growth factor agent aflibercept.

	Total (N = 48)	Recurrence group (n = 37)	No-recurrence group (n = 11)	*P* value
Age, years, mean (SD)	67.4 (9.4)	68.3 (9.5)	64.4 (8.5)	0.228
Male, n (%)	19 (39.6)	12 (32.4)	7 (63.6)	0.063
Female, n (%)	29 (60.4)	25 (67.6)	4 (36.4)	
Preclinical period, d, mean (SD)	41.9 (27.4)	38.6 (25.8)	53.1 (30.7)	0.121
BCVA, logMAR				
Mean (SD)	0.37 (0.25)	0.37 (0.24)	0.35 (0.31)	0.743
Range	0–1.0	0.1–1.0	0.1–1.0	
CMT, mm, mean (SD)	625 (190)	656 (185)	520 (176)	0.036

*BCVA* best corrected visual acuity, *CMT* central macular thickness, *logMAR* logarithm of the minimum angle of resolution.

*P* value from 1-way analysis of variance.

### Clinical course after aflibercept injection

**Figure 1:**
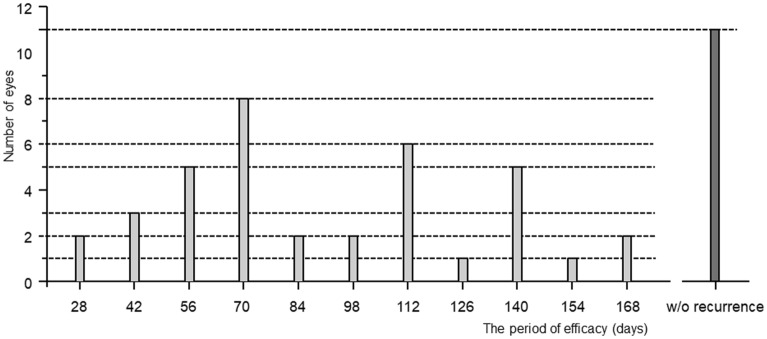
The number of eyes according to the period of efficacy. The period of efficacy appeared to show a bimodal pattern, with a group of participants showing recurrence before 12 weeks and one showing recurrence after 16 weeks. Eleven eyes showed no recurrence.

After the initial injection of aflibercept, BCVA improved over time in the 37 eyes with recurrence and reached its highest level of 0.02 ± 0.15 logMAR after 45.5 ± 28.1 days. CMT also gradually improved in these eyes and decreased to 274 ± 38 mm after 45.5 ± 20.6 days. The mean period of efficacy (i.e., the period between the first and second injections) was 92.5 ± 40.8 days, and the mean number of visits was 7.6 ± 2.9. However, both varied greatly (ranges, 28–196 days and 3–15 visits, respectively), and the period of efficacy appeared to show bimodal peaks (Fig. 1). After the initial injection of aflibercept, BCVA improved over time in the 11 eyes with no recurrence and reached its highest level of − 0.06 ± 0.08 logMAR after 47.0 ± 49.4 days. CMT also gradually improved in these eyes and decreased to 270 ± 26.5 mm after 68.6 ± 45.2 days.

**Figure 2:**
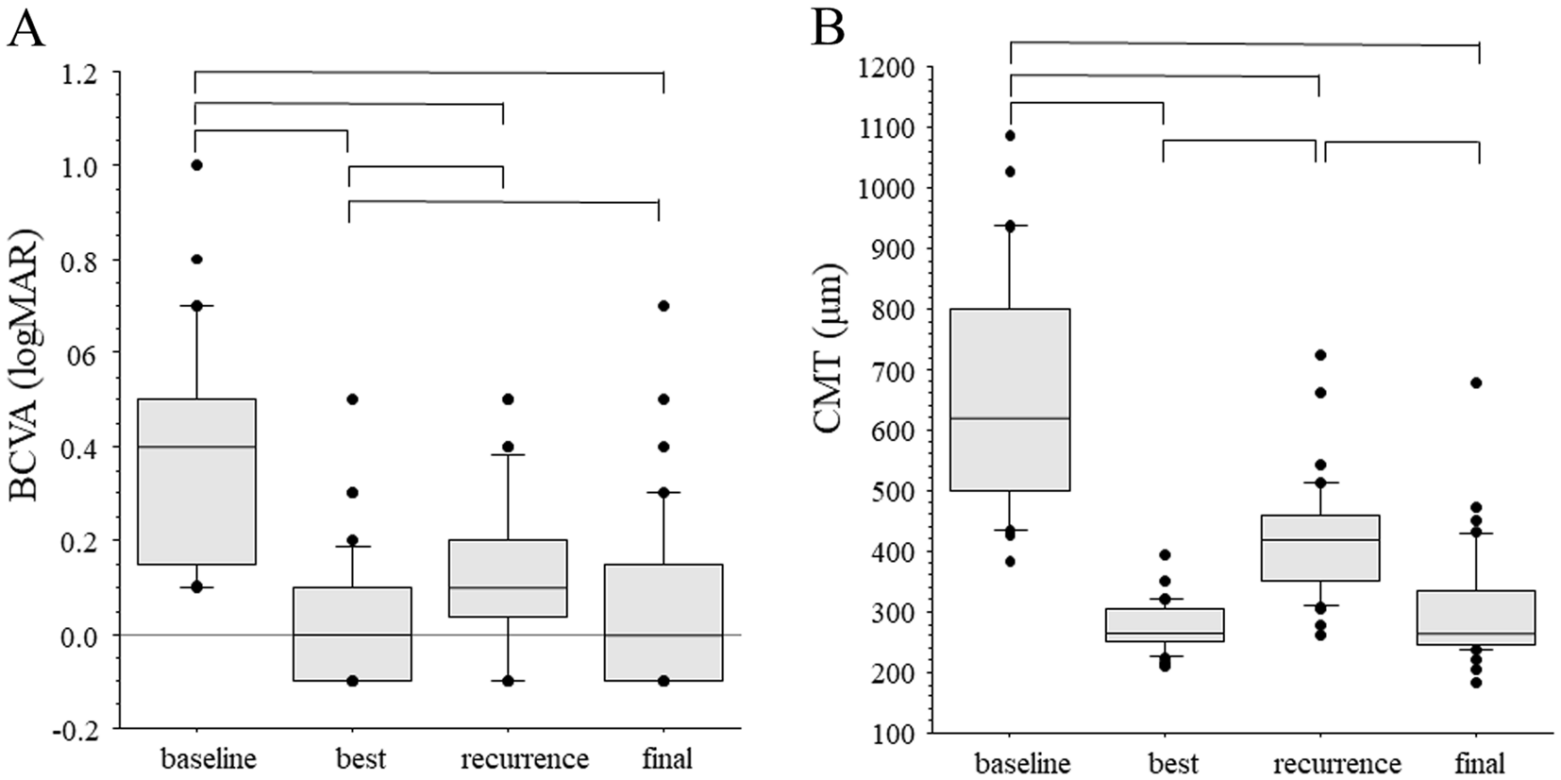
(**A**) Best corrected visual acuity (BCVA) at baseline, best BCVA in period of efficacy, and BCVA at first recurrence and at end of study period (24 months). (**B**) Central macular thickness (CMT) at baseline, best CMT in period of efficacy, and CMT at first recurrence and at end of study period (24 months). Data range in box and whisker plots, 25% to 75% and 10% to 90%; center line, median; each dot, outlier (i.e., < 10% or > 90%).

After 24 months, BCVA had significantly improved from baseline (from 0.37 ± 0.25 to 0.07 ± 0.18 logMAR, *p* < 0.001), as had CMT (from 625 ± 190 to 304 ± 96 mm; *p* < 0.001). Patients showed a significant decrease in BCVA from the best value in the period of efficacy to the value at the 24-month visit (*p* = 0.044), but no such difference was found for CMT (*p* = 0.060; Fig. 2). In no-recurrence group (n = 11), patients showed no significant decrease in BCVA from the best value in the period of efficacy (− 0.06 ± 0.08 logMAR) to the value at the 24-month visit (− 0.04 ± 0.15 logMAR; *p* = 0.341) and no such difference was found for CMT (best value, 270 ± 26.5 mm; final value, 285 ± 36.6 mm; *p* = 0.131).

The total mean number of aflibercept administrations in the eyes showing recurrence (n = 37) was 7.2 ± 2.9 (range, 2–14), and these patients required a mean of 15.8 ± 3.4 visits (period of efficacy, 7.6 ± 2.9 visits; after second aflibercept administration, 8.2 ± 4.5 visits). In the no-recurrence group (n = 11), these patients required a mean of 14.9 ± 1.22 visits (period of efficacy, 12.5 ± 0.69 visits; after second aflibercept administration, 2.4 ± 0.67 visits).

### Order of change in BCVA and CMT during the period of efficacy

**Figure 3:**
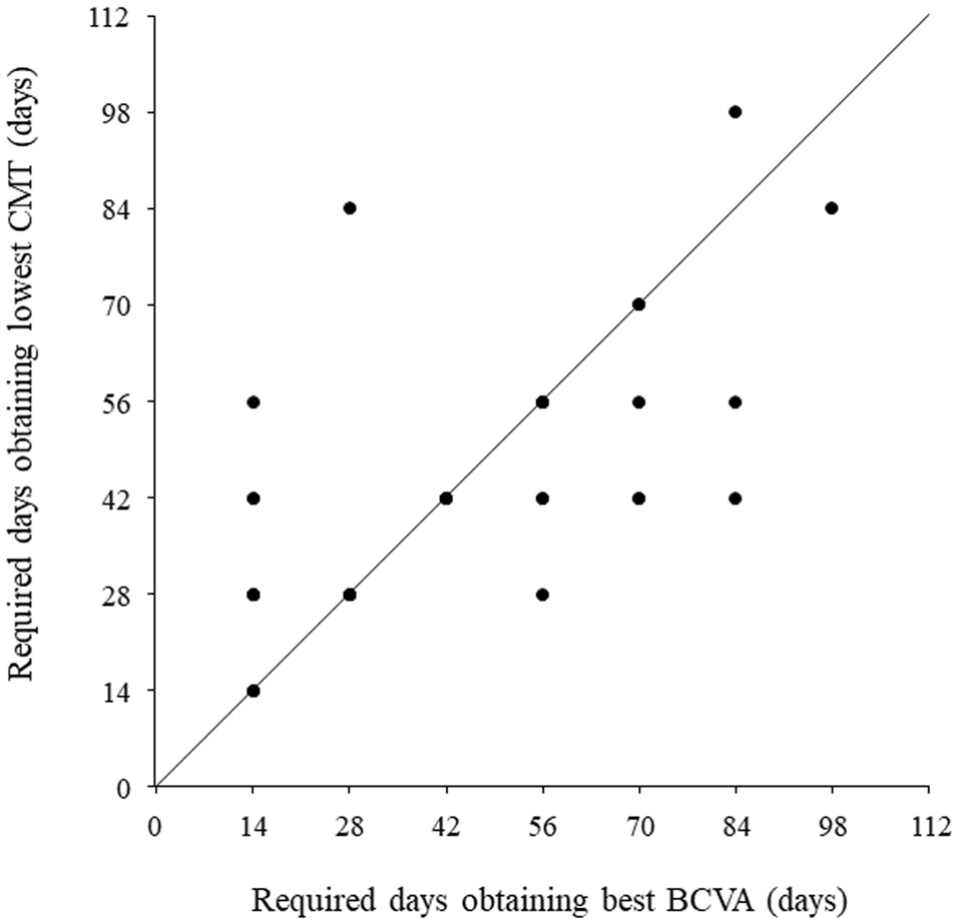
Time in period of efficacy (in days) until highest values of best corrected visual acuity (BCVA) and smallest value of central macular thickness (CMT). The theoretical line reflects the days with the highest BCVA value and the smallest CMT value.

The temporal relationship between the best BCVA and best CMT in each eye at the monitoring visits every 2 weeks are shown in Fig. 3. The best BCVA occurred before the best CMT in 10 of the 37 eyes with recurrence (27.0%), and the best CMT occurred before the best BCVA also in 10 eyes (27.0%). BCVA at baseline and after 24 weeks showed no significant difference between these 2 groups, but the former group had a significantly shorter time between the first symptoms and the first ophthalmological examination (best BCVA first, 23.0 ± 11.9 days; best CMT first, 50.7 ± 25.5; *p* = 0.006).

## Discussion

This 24-month study investigated individualized treatment regimens with the anti-VEGF agent aflibercept in untreated patients with macular edema due to BRVO. VEGF concentrations vary from person to person^7^, so the period of efficacy of aflibercept is expected also to vary among individuals. To assess the efficacy of aflibercept, the current study assessed the period of efficacy, defined as the period from the first administration of aflibercept to recurrence of macular edema. In case of recurrence, a second aflibercept injection was administered and an individual treatment interval was defined for each patient as 1 week less than the period of efficacy. If necessary, the treatment interval was adjusted over the 2-year study period. Macular edema recurred in 37 of 48 eyes in 48 patients. These patients received several aflibercept injections according to their personalized treatment regimens and showed a visual gain after 24 months, supporting the benefit of our novel protocol. The period of efficacy, which reflected treatment response to aflibercept, ranged from 4 to 24 weeks and showed a bimodal pattern in that recurrence occurred within 12 weeks in one group of patients and after 16 weeks in another.

Several RCTs on anti-VEGF treatment for BRVO with macular edema have studied unique treatment regimens composed of an induction and maintenance phase. In the BRIGHTER study^3^, a 3 + pro re nata (PRN) protocol with ranibizumab resulted a gain of 15.5 letters after 2 years. The HORIZON (BRVO) study^2^, which was an extension of the BRAVO study, found that a 6 + PRN protocol with ranibizumab resulted in a 17.5 letters gain after 2 years. The mean number of injections of anti-VEGF drugs required over 2 years in each study was follows: BRIGHTER, 11.4; and HORIZON (BRVO), 10.5. In the HORIZON (BRVO) study^2^, the significant visual improvements achieved with the first continuous injections were generally maintained up to 12 months by subsequent monthly monitoring and PRN maintenance injections; however, reduced monitoring frequency in the second year resulted in a decrease in the visual improvements. The real-world study found a 13.7-letter gain at 2 years after 5.8 injections of aflibercept^6^, which was less improvement with fewer injections than in the RCTs.

In our protocol, during the induction phase we evaluated response of macular edema associated with treatment-naïve BRVO by monitoring patients every 2 weeks after an initial injection of aflibercept; a mean of 7.6 ± 2.9 visits occurred during the period of efficacy, i.e., before recurrence of edema. After recurrence, visits and injections were performed on the basis of the individual treatment interval, which was 1 week less than the period of efficacy, and the treatment interval was adjusted during the maintenance phase on the basis of drug response. In this study, three of 37 patients in the recurrence group had relapsed at the time of the personalized treatment interval. The low recurrence rate (10% or lower) indicates that it may not be necessary to choose a closer follow-up; however, further study is needed to investigate the relationship between the individual treatment interval and the recurrence of macular edema.

The 2-year visual outcome of a 15.7 letter gain in our study was almost the same as those in the other RCTs described above. However, our patients required a mean of 7.2 injections of aflibercept, which is less than in the above-mentioned RCTs. The present study did not include an induction phase with continuous administrations of the anti-VEGF agent and instead adopted the novel approach of assessing response by examining treated eyes every 2 weeks. Unclear is whether several continuous monthly injections are required as a treatment induction phase in every eye with BRVO and macular edema. In clinical practice, we sometimes encounter patients with BRVO and macular edema who achieve complete resolution of macular edema with a single injection of an anti-VEGF drug. A previous study found no difference of visual outcome between 1 + PRN and 3 + PRN protocols in eyes with BRVO and macular edema^8^, although the study was a small case series and had some limitations.

In this study, we found no recurrence of edema within 24 months in 11 of 48 patients; this ratio was equivalent to the one found in our previous study^9^, which showed that 12 of 46 patients had no recurrence when treated by a 1 + PRN procedure with ranibizumab. Furthermore, in the present study, baseline CMT was significantly lower in the no-recurrence group than in the recurrence group; this finding is also supported by our earlier study^9^, which found that baseline CMT was significantly correlated with the number of IRI injections. Moreover, the reduction in macular vessel density was found to be significantly and negatively correlated with the number of IRI injections^10^. In addition, we reported previously that baseline aqueous humor levels of inflammatory cytokines (soluble intercellular adhesion molecule-1 and interleukin-6 and -8) were significantly correlated with the number of IRI but not VEGF injections^9^. Therefore, recurrent macular edema may be related to various factors, such as macular vessel density or levels of inflammatory cytokines. In the future, we would like to use the present protocol to investigate the time course of both macular vessel density and levels of inflammatory cytokines.

We found that in 27.0% of eligible eyes the best BCVA was measured prior to the best CMT in the period of efficacy and, in those eyes, the length of time between the first symptoms and the first ophthalmological examination was shorter than in the group with the best CMT prior to the best BCVA. A previous study indicated that the post-treatment relationship between change of BCVA and change of CMT seems to show a higher correlation in retinal vein occlusion (RVO) than in diabetic macular edema and macular edema associated with noninfectious uveitis^11^. The pathogenesis of macular edema due to RVO is different from that due to diabetic retinopathy and noninfectious uveitis in that in RVO it is caused by an acute breakdown of the blood retinal barrier in the branch retinal vein and subsequent fluid accumulation and tissue swelling^12^. The size of the ischemic area, amount of inflammation, and duration of tissue swelling may play a role in the photoreceptor damage associated with macular edema^13^, and these variables may worsen over time if treatment for macular edema is delayed, resulting in permanent vision impairment. A retrospective chart review showed that a longer period between the start of symptoms and the first anti-VEGF treatment was associated with less improvement in letters read after both 6 and 12 months and that neovascular and total events were more common if the time was 30 days or longer^14^. The duration of the preclinical period did not influence the visual prognosis in our study participants, but visual improvement was seen before morphological recovery in the eyes that were treated after a shorter preclinical period. Eligible eyes in this study were limited to treatment-naïve, non-ischemic cases; therefore, even eyes that showed incomplete morphological recovery of the edema may have obtained functional recovery of vision earlier. Further investigations are required to clarify this hypothesis.

Although our novel protocol was found to be useful and effective and was not inferior to routine protocols evaluated in previous RCTs, our study has several limitations. Eligible eyes were limited to relatively short preclinical periods (less than 3 months). Furthermore, we included a relatively small number of cases. Nevertheless, we found that response to the anti-VEGF drug aflibercept in eyes with BRVO and macular edema varied greatly between patients. Thus, individualized protocols should be considered for the treatment of macular edema associated with BRVO.

## Patients and methods

### Ethics statement

This study followed the tenets of the Declaration of Helsinki, with approval for this study obtained from the Ethics Committee of Tokyo Medical University Hachioji Medical Center (IRB No. H-173). All procedures performed in studies involving human participants were in accordance with the ethical standards of the institutional and/or national research committee and with the 1964 Helsinki declaration and its later amendments or comparable ethical standards. Informed consent was obtained from all individual participants included in the study. This study was registered in the University Hospital Medical Information Network (UMIN) clinical trials registry (UMIN000030311; 12/9/2017).

### Patients

Patients with treatment-naïve BRVO with visual impairment due to macular edema were eligible to participate in this prospective, open-label study. Participants were recruited from among all consecutive patients diagnosed with macular edema due to BRVO at the Department of Ophthalmology, Hachioji Medical Center, Tokyo Medical University, Tokyo, Japan. Inclusion criteria were age, 41 years or older; length of period between first symptoms and first ophthalmological examination, shorter than 3 months; best corrected visual acuity (BCVA), 0.1 to 1.3 logarithm of the minimum angle of resolution (logMAR; Early Treatment Diabetic Retinopathy Study letters, 20 to 80); and central macular thickness (CMT) assessed with optic coherence tomography (OCT), more than 300 µm.

### Exclusion criteria

Eyes with macular ischemia were excluded from the study, as were eyes that had been previously treated, e.g., with laser treatments, vitrectomy, or drug injections. Patients with systemic disorders other than hypertension or hypercholesterolemia were also excluded. Patients with any of the following in their medical history were excluded: glaucoma, uveitis, other retinal disease, ischemic-type retinal ischemia, type 2 diabetes, rubeosis iridis, eye infections, treatment by laser photocoagulation, and cataract or other intraocular operation. Eyes with macular BRVO, defined as the macular subtype according to the site of the occlusion, also were excluded.

### Clinical parameters

All patients received a comprehensive ocular examination before the start of the study and at every follow-up visit. Fluorescein angiography was performed in each patient at the initial visit to determine the area of capillary non-perfusion. During the follow-up examinations, visual acuity was measured with a logMAR chart (5 m; NEITZ LVC-10, Tokyo, Japan) and CMT was recorded at the central 1-mm field in mapping images generated by Spectralis OCT (Heidelberg Engineering, Heidelberg, Germany).

### Study design (injection protocol)

Initially, all eligible eyes were injected with 0.05 ml of 2.0 mg aflibercept (Eylea, Bayer HealthCare Pharmaceuticals in Japan, Osaka, Japan) into the vitreous cavity with a sharp 30G needle at a distance of 3.5 mm from the limbus. After receiving the first injection of aflibercept, participants were instructed to return to the hospital every 2 weeks for the duration of the treatment period (24 weeks) for assessment of recurrence, defined as a decrease in BCVA and increase in CMT compared with the previous values. In case of recurrence, aflibercept injection was repeated and the period of efficacy was recorded as the period since the first injection. The treatment interval for each patient was then determined by subtracting 1 week from the period of efficacy^15^. Patients who showed recurrence and received a repeat injection of aflibercept subsequently visited the hospital according to their individualized treatment interval and received a repeat injection aflibercept at each visit. Macular edema was assessed at each study visit, and the treatment interval was decreased by a week if edema had worsened or increased by a week if it had improved. In this study, worsening of edema was defined as a CMT of greater than or equal to 300 μm, and improvement, as a CMT less than 300 μm. This individualized treatment protocol was applied in each participant^15^, and participants were assessed from the first injection of aflibercept for 24 months.

### Outcome measures

The main outcome measures were the difference between the 24-month BCVA and both the baseline BCVA and the best BCVA during the period of efficacy. Additional outcome measures were the period of efficacy, and the number of visits and number of intravitreal aflibercept administrations in the 24-month study period. As a secondary outcome measure, we assessed the dynamic change of CMT.

### Statistical analysis

Data are shown as mean ± SD. Changes in the same eyes were compared with Student’s paired *t* test, and changes in different eyes, with the Mann–Whitney *U* test. Statistical significance was assumed at a 2-tailed *p* value below 0.05. An independent center (STATZ Institute, Tokyo, Japan) performed all analyses with the software SAS 9.4 (TS1M5; SAS Institute, Cary, NC, USA).

## Data Availability

The datasets used and/or analyzed during the current study are available from the corresponding author on reasonable request.
